# Functional characterization of the protein C A267T mutation: evidence for impaired secretion due to defective intracellular transport

**DOI:** 10.1186/1471-2121-11-67

**Published:** 2010-09-06

**Authors:** Lena Tjeldhorn, Nina Iversen, Kirsten Sandvig, Jonas Bergan, Per Morten Sandset, Grethe Skretting

**Affiliations:** 1 Department of Hematology, Oslo University Hospital, Oslo, Norway; 2 Department of Medical Genetics, Oslo University Hospital, Oslo, Norway; 3 Department of Biochemistry, Institute for Cancer Research, The Norwegian Radium Hospital, Oslo University Hospital, Oslo, Norway; 4 Institute of Clinical Medicine, University of Oslo, Oslo, Norway; 5 Centre for Cancer Biomedicine, University of Oslo, Oslo, Norway

## Abstract

**Background:**

Activated protein C (PC) is a serine protease that regulates blood coagulation by inactivating coagulation factors Va and VIIIa. PC deficiency is an autosomally inherited disorder associated with a high risk of recurrent venous thrombosis. The aim of the study was to explore the mechanisms responsible for severe PC deficiency in a patient with the protein C A267T mutation by *in-vitro *expression studies.

**Results:**

Huh7 and CHO-K1 cells were transiently transfected with expression vectors containing wild-type (WT PC) and mutated PC (A267T PC) cDNAs. PC mRNA levels were assessed by qRT-PCR and the PC protein levels were measured by ELISA. The mRNA levels of WT PC and A267T PC were similar, while the intracellular protein level of A267T PC was moderately decreased compared to WT PC. The secretion of A267T PC into the medium was severely impaired. No differences in molecular weights were observed between WT and A267T PC before and after treatment with endo-β-N-acetylglucosaminidase. Proteasomal and lysosomal degradations were examined using lactacystin and bafilomycin, respectively, and revealed that A267T PC was slightly more susceptible for proteasomal degradation than WT PC. Intracellular co-localization analysis indicated that A267T PC was mainly located in the endoplasmic reticulum (ER), whereas WT PC was observed in both ER and Golgi.

**Conclusions:**

In contrast to what has been reported for other PC mutants, intracellular degradation of A267T PC was not the main/dominant mechanism underlying the reduced intracellular and secretion levels of PC. Our results indicate that the A267T mutation most likely caused misfolding of PC, which might lead to increased retention of the mutated PC in ER.

## Background

Protein C (PC) is a vitamin-K dependent zymogen, which upon activation to a serine protease, plays an important role in the regulation of blood coagulation through the inactivation of factors Va and VIIIa [1]. PC deficiency is an autosomally inherited disorder associated with increased risk of venous thrombotic complications, such as deep vein thrombosis and pulmonary embolism [2,3].

Human PC is synthesized as a 461 amino acid single polypeptide chain that undergoes extensive post-translational modifications including signal peptide cleavage, γ-carboxylation, β-hydroxylation, and N-linked glycosylation before it is secreted by the liver [4]. PC circulates in the plasma in several glycoforms and it has been shown that glycosylation of human PC affects its secretion, processing and antithrombotic activities [5]. A wide variety of genetic mutations in the PC gene (PROC) have been shown to be associated with PC abnormalities http://www.itb.cnr.it/procmd/. Most of these are missense mutations although a few nonsense and frameshift mutations, or splice-site abnormalities have been reported as well [6]. Several *in vitro *expression studies have investigated the molecular mechanisms of mutations in the PROC gene associated with PC deficiency. Results from these studies indicated that mutated PC variants were secreted inefficiently from transfected cells compared to wild-type (WT) PC [7-15]. Some of the studies also demonstrated that the intracellular levels of the mutated PC were decreased compared to WT PC, suggesting increased intracellular degradation of the mutated PC to be a dominant pathway behind the impaired secretion [8,10,11,15].

In eukaryotic cells, intracellular degradation of mutated proteins is known to be carried out by two main proteolytic pathways, namely endoplasmic reticulum (ER) associated degradation (ERAD) (through proteasomes) or autophagy (through lysosomes) [16]. Most secretory proteins first enter the ER where they are subjected to post-translational modifications and folding prior to their transit to Golgi and subsequent to the cell surface [17,18]. Only properly modified and folded proteins are supposed to exit the ER. Most misfolded proteins are retained within the ER lumen in complex with molecular chaperones, then retrogradely transported to the cytosol and eventually degraded through the proteasomes [15,19-22]. Misfolded proteins not transported to the cytosol may aggregate transiently or permanently in ER [17]. Accumulation of misfolded proteins in ER can cause ER stress and activation of a protective response known as unfolded protein response (UPR), which implicate three different mechanisms to restore homeostasis: attenuation of protein synthesis, optimization of chaperone-assisted protein folding and activation of protein degradation [23]. Several studies have revealed that protein degradation in ERAD can be compromised under ER stress resulting in insufficient proteasomal degradation [24,25]. The mechanisms associated with the intracellular processing of mutant proteins are complex and sorting of proteins for ERAD remains poorly understood. Criteria such as molecular chaperones, conformation and folding factors are most likely involved in targeting of mutated proteins for degradation [26,27]. Previous studies have shown that mutations in the PC molecule caused PC deficiency due to impaired transport of PC from ER [7,10,13] and some of the studies also detected increased degradation by proteasomes [15,20,28].

The aim of the present work was to characterize the A267T PC mutation previously reported in a patient with PC deficiency [29]. Using site-directed mutagenesis to generate A267T PC cDNA and subsequent transient transfections, we explored the potential molecular mechanism(s) by which this mutation may cause a reduction of the PC level in the plasma of the patient. It should be emphasized that results obtained with *in-vitro *studies after overexpression of proteins, may differ from the *in-vivo *situation. However, it is likely that the potential mechanisms involved will be revealed. We found that the A267T PC levels in transfected cells and culture medium were strongly reduced compared to the level of WT PC despite of no differences in the steady state levels of the A267T and WT PC mRNA. Similar to other PC mutants, the A267T PC had impaired intracellular transport, however, only a small fraction of the A267T PC was degraded by the proteasomes.

## Results

### Transient expression of WT and A267T PC

**Figure 1:**
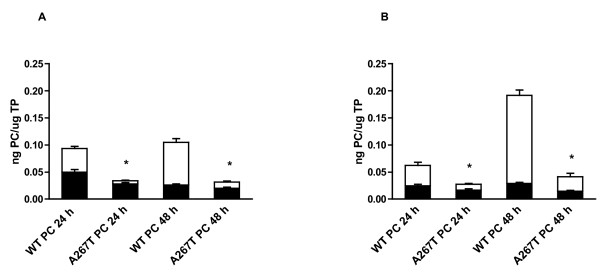
**Reduced level of A267T PC in transiently transfected cells**. CHO-K1 (A) and Huh7 (B) cells were transiently transfected with plasmid constructs expressing WT or A267T PC. The culture medium ('white square') and cell lysates ('black square') were harvested 24 and 48 h after the transfection. The PC levels were measured using an ELISA kit and the PC levels in Huh7 were adjusted to the endogenous PC levels in these cells as described in Methods. The concentration of PC was adjusted to TP concentration in the corresponding sample. Histograms and the bars represent the mean and SEM values for CHO-K1 (n = 9) and for Huh7 cells (n = 8). * *p *< 0.05 (Mann-Whitney test, A267T PC versus WT PC).

**Figure 2:**
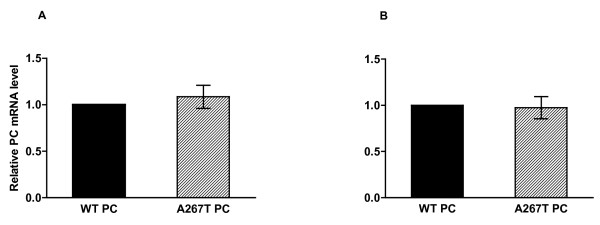
**No differences in mRNA levels between WT and A267T PC**. CHO-K1 (A) and Huh7 cells (B) were transiently transfected with plasmid constructs expressing WT or A267T PC. Cells were harvested 24 h after transfection and total RNA was isolated. PC mRNA levels were determined using quantitative RT-PCR with 18 S and GAPDH as endogenous controls for CHO-K1 and Huh7 cells, respectively. The mRNA PC levels i Huh7 cells were normalized for the endogenous mRNA PC levels. The results are presented as ratio between mRNA levels of WT and A267T PC with the WT PC level assigned as 1. The results are from three independent transfection experiments performed in triplicates and presented as mean ± SEM.

To investigate the effect of the A267T mutation on the synthesis and secretion of PC, we transiently transfected CHO-K1 and Huh7 cells with WT PC or A267T PC cDNA constructs. Assessment of PC levels in the cell lysates and the corresponding medium revealed that the total amount of the A267T PC mutant was severely reduced in CHO-K1 cells (Figure 1A). The intracellular levels of A267T PC were significantly lower, 57 and 77% relative to the WT PC levels 24 and 48 h after transfection, respectively. A dramatic reduction in the amount of secreted A267T PC was detected, 14 and 16% of the WT PC secretion levels 24 and 48 h after transfection, respectively. Similar results were obtained in Huh7 cells, which have endogenous expression of protein C (Figure 1B). The corresponding results were 66 and 50% of the intracellular WT PC levels and 29 and 17% of the secreted WT PC levels. The results strongly indicate that the mutation affects both the synthesis and the secretion of the protein. No PC antigen was detected in CHO-K1 cells transfected with empty vector. All results for PC concentration were adjusted for total protein (TP) concentration but similar results for PC expression were also obtained for PC concentration not adjusted for TP. We then examined whether the reduced total amount of A267T PC was due to differences in the mRNA steady state levels of the two PC variants. The mRNA expression levels of WT and A267T PC were determined by quantitative RT-PCR in transiently transfected cells. No differences in the PC mRNA levels were detected between A267T PC and WT PC either in CHO-K1 or in Huh 7 cells (Figure 2A, B).

### N-linked glycosylation and Western analysis

**Figure 3:**
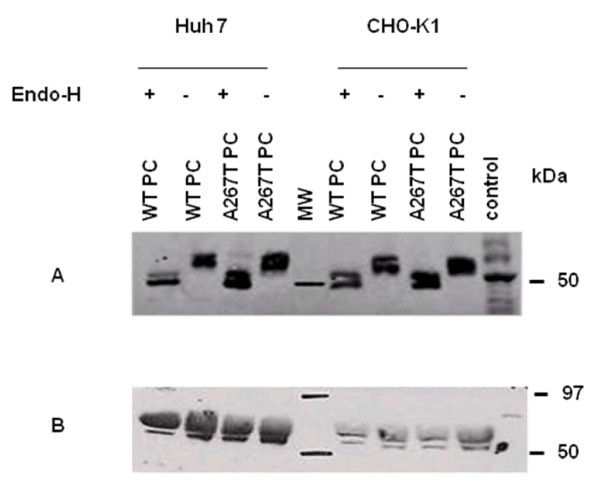
**N-linked glycosylation and Western analysis**. Lysates (A) and culture medium (B) from Huh7 and CHO-K1 cells transiently transfected with plasmid constructs expressing WT or A267T PC were harvested 48 h after transfection and treated ± Endo-H for 18 h at 37°C. After completion of the reaction, the samples were analyzed by 10% SDS-PAGE under reducing conditions. Following blotting onto a PVDF membrane and subsequent incubation with antibody against PC, the bands were visualized with ECL. Equal amounts of PC (as measured by ELISA) in cell lysates and culture medium were analyzed.

Protein C has four potential sites for N-glycosylation. It has previously been shown for other PC mutants that their destiny was dependent on N-linked glycosylation [28]. Thus, a possibility existed that the reduced intracellular levels and secretion of A267T PC could be due to differences in N-linked glycosylation between the two protein variants. Lysates from cells transiently transfected with plasmid constructs expressing WT or A267T PC, were treated with Endo-H enzyme followed by Western blot analysis (Figure 3A). The molecular weights of the two bands of WT and A267T PC were equally reduced compared to lysates from untreated cells. The molecular weights of secreted WT and A267T PC were the same for both variants and importantly not affected by Endo-H (Figure 3B). This indicated that only fully processed PC molecules were secreted. As seen for the untreated samples, the A267T mutation did not affect the size of PC since no differences in the molecular weights were observed between the mutant and WT PC.

### Stability and degradation of WT and A267T PC

Since no differences in the steady state levels of mRNA for the two PC variants were observed, the reduced intracellular level of A267T PC compared to the WT level could be explained by alterations in stability of the mutated PC. To determine the half-lives of the two PC variants, transient transfected CHO-K1 cells were treated with the protein synthesis inhibitor cycloheximide (CHX) for various time-points. The half-lives were found to be five and six hours (h) for WT and A267T PC, respectively (data not shown). In fact, this indicated that the mutant was slightly more stable than the WT PC. Therefore, we aimed to examine whether the differences in intracellular protein levels could be due to changes in degradation. Transiently transfected CHO-K1 cells were treated with the proteasomal degradation inhibitor lactacystin or the lysosomal degradation inhibitor bafilomycin (Table 1). Treatment with lactacystin resulted in a modest, although statistically significant (*p *= 0.045) difference between the PC variants. WT and A267T PC levels were elevated (19.8% and 31.8%, respectively) compared to the intracellular PC levels in corresponding untreated cells, suggesting a slight increase in proteasomal degradation of A267T PC compared to WT PC. The bafilomycin treatment resulted only in a non-significant minor increase of both WT PC (12.6%) and A267T PC (6.7%) intracellular levels, and indicated no lysosomal degradation of the PC variants (Table 1).

**Table 1:** Effect of protein degradation inhibitors on intracellular PC levels.

Treatments	Intracellular WT PC (%)	Intracellular A267T PC (%)
Control	100	100

Lactacystin (10 μM), n = 8	119.8 ± 3.3*	131.8 ± 4.4*

Bafilomycin (100 nM), n = 9	112.6 ± 4.9	106.7 ± 7.2

Transient transfected CHO-K1 cells expressing WT or A267T PC were incubated with medium containing 10 μM lactacystin or 100 nM bafilomycin for 24 and 8 h, respectively. PC protein levels were measured in cell lysates and results are calculated as percentage of controls (samples not treated with protein degradation inhibitors). Data are presented as mean ± SEM. *p < 0.05 (Wilcoxon test).

### Intracellular localization of WT and A267T PC

**Figure 4:**
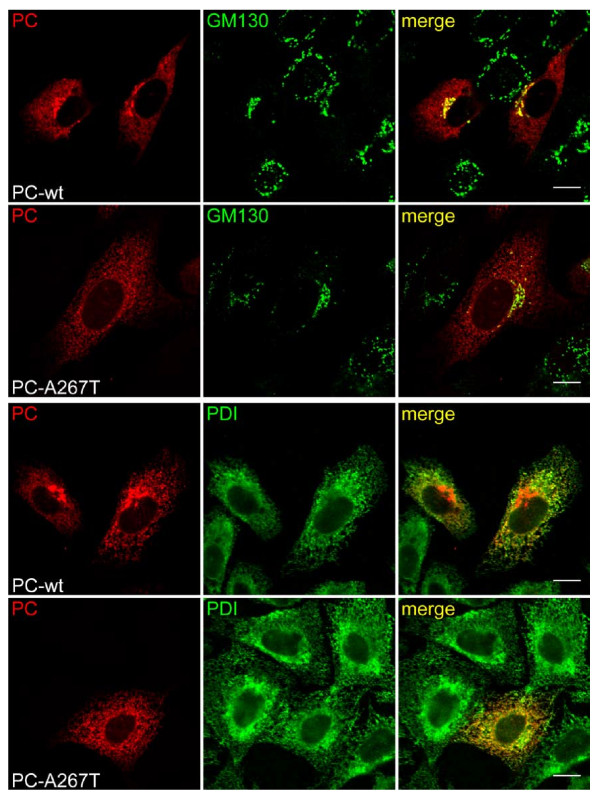
**Intracellular localization of WT and A267T PC**. CHO-K1 cells were grown on coverslips and transiently transfected with plasmid constructs expressing WT or A267T PC. Forty-eight h after transfection the cells were fixed and stained with anti-PC and anti-PDI (ER marker) or anti-GM130 (Golgi marker). PC appears red, PDI and GM130 appears green and overlaid images is yellow and corresponds to areas of co-localization of PC with ER and Golgi. Scale bar, 10 μm.

Since no major differences in stability and degradation between WT and A267T PC could account for the low levels of the mutated protein, we speculated whether the two variants might have different intracellular distribution. It has previously been reported that abnormal protein C molecules were retained in the ER [7,10,13]. We therefore examined the localization of PC in transiently transfected CHO-K1 cells by immunofluorescence analysis. In order to explore the location of WT and A267T PC, the cells were doublestained with antibodies against PC and either the ER marker PDI or the Golgi marker GM130. Figure 4 demonstrates that both WT and A267T PC co-localized with PDI and GM130 while the A267T PC co-localized with GM130 in a much less extent than did WT PC. These results indicated that A267T PC was predominantly localized in the ER and transported to the Golgi with less efficiency compared to WT PC.

## Discussion

The present study provides evidence that the mechanism of PC deficiency caused by the A267T mutation [29] most likely involves impaired synthesis and also reduced secretion of the mutant. In contrast to what has been demonstrated for several other PC mutations causing PC deficiency [15,20,28], the effect of the present mutation on proteasomal degradation was minimal. However, like other PC mutations [7,10,13], the A267T variant was retained in the ER. Transient expression analysis demonstrated that the A267T PC protein levels secreted into the culture medium were seven and five fold less compared to WT PC in CHO-K1 and Huh7 cells, respectively. The intracellular levels of the A267T PC were also strongly reduced. No major differences in mRNA levels, half-lives or degradation were detected for the two PC variants. Immunofluorescence staining revealed that the mutant was observed mainly in ER while WT PC was detected both in ER and in Golgi indicating a rapidly processing and secretion of WT PC, while the majority of mutated PC most likely was retained in the cells in an attempt to be folded correctly.

PC deficiency is an inherited disorder associated with a high risk of recurrent venous thrombosis [2,3]. *In vitro *studies of various mutations in the PROC gene have provided insight into how these mutations can cause PC deficiency [7-15,20,28]. Most of these studies revealed that the PC deficiency was due to impaired secretion caused by intracellular degradation of the mutated proteins [8,10,11,15,20,28]. Enhanced proteolytic degradation of mutated proteins is a common molecular pathological mechanism in many diseases [30]. Degradation is often associated with impaired intracellular transport of proteins in the producing cells [31]. Intracellular protein transport through the secretory pathway in eukaryotic cells depends on a proper folding of proteins in ER [17,18]. Several transfection studies have described impaired transport of proteins from ER to Golgi due to prolonged binding of the mutated proteins with chaperones which facilitate a folding process in ER [7,32]. Mutated/misfolded proteins are frequently subjected to subsequent degradation by quality control mechanisms involving ERAD [17]. Not all mutated proteins will be recognized as misfolded by the ER quality control system. Some of them can remain in ER for an extended time period without being sent for disposal in ERAD [24,33-35]. It can be speculated whether the effect a mutation has on 3 D structure of a protein can determine which mechanism that will be chosen in each particular case. It is likely that mutations, which are able to cause severe conformational changes, trigger ERAD to become hyperactive resulting in nearly complete degradation of the mutated proteins whereas a mutation with minor effects on the protein structure can result in several rounds of the folding process and thereby increased ER retention and attenuated degradation. The latest database on 3D-structure of PC http://www.itb.cnr.it/procmd/, obtained by computational approaches, interpreted the A267T mutation as not being disruptive for the structure and function of PC. In addition, alignment of the PC amino acid sequence and other evolutionary related human vitamin-K-dependent factors revealed that the A267 is not strictly conserved. All these facts may indicate that the investigated mutation leads to only minor effects on the PC molecule and inefficient ERAD degradation.

The fact that the intracellular level of A267T PC was reduced and the mutated PC was not degraded faster compared to WT PC could indicate that mRNA translation might be affected as indicated by other reports [32,36]. The most immediate response to ER stress, which can occur as a result of misfolded protein accumulation in ER, is transient attenuation of mRNA translation which prevents influx of newly synthesized polypeptides in ER [16,23]. Although no evidence of ER stress is presently available, one might speculate that reduced mRNA translation of mutated PC as a result of UPR could explain the fact that the intracellular levels of A267T PC was reduced compared to the WT PC levels. *In-vitro *translation experiments might have revealed if this was the case. Since the effect of inhibition of proteasomal degradation in this study was found to be insufficient to explain the reduced intracellular and secreted levels of A267T PC, the involvement of additional degradation pathways can not be ruled out. A small number of mutated secretory proteins have been reported to be degraded both by proteasomes and other cytosolic proteases (often serine/cysteine) simultaneously [37-39].

In some of the previous studies where the proteasomal degradation was found to be the dominant mechanism of PC deficiency [15,20,28], it was shown that this degradation was independent of ubiquitinylation of mutated PC. Ubiquitinylation is essential in several cellular processes including targeting proteins for proteasomal degradation and is catalyzed by the sequential action of various enzymes [40]. In our study, we found that inhibition of ubiquitinylation by pyrazone (PYR-41) had no effect on the PC levels of either WT or A267T PC (data not shown) and indicated that proteasomal degradation of the investigated PC variants was independent of ubiquitin.

Intracellular protein transport through the secretory pathway in eukaryotic cells consists of translocations from ER to Golgi and from Golgi to secretory vesicles [41]. Like other secretory glycoproteins, PC undergoes post-translational processing such as glycosylation, γ-carboxylation and β-hydroxylation. N-linked glycosylation of PC takes place before transport to the Golgi apparatus and possibly as a co-translational event in ER. On their way through the Golgi, the carbohydrate side chains of PC undergo further modifications which lead to resistance against Endo-H treatment of the protein in Golgi [42]. The Endo-H digestion analysis in the present study revealed that both WT and A267T PC proteins in the cell lysates were sensitive to the Endo-H treatment, indicating that the PC was localized predominantly to the ER. However, the immunofluorescence analysis in this study demonstrated that a fraction of WT PC was located in Golgi, and thus, we expected some of the cellular WT PC to be resistant to Endo-H. This was however, not the case. In agreement with other studies [10,15] we therefore assume that most WT PC resided in the ER and was secreted quickly subsequent to post-translational modifications.

## Conclusions

In the present study we have transiently transfected cells with cDNAs encoding WT and A267T PC and found that the mutation led to reduced intracellular levels and impaired secretion of the mutant protein relative to the WT PC. The level of secreted PC mutant was in agreement with the observed plasma PC protein level in our patient who had 12% of the normal level [29]. The differences in protein levels of the two PC variants were not due to reduced transcription of the mutated gene. The A267T PC was mainly localized in the ER and presumably transported to Golgi with less efficiency compared to WT PC. This could account for the reduced secretion of the mutant. Since the reduced intracellular levels and secretion of A267T PC could not be explained by differences in proteasomal or lysosomal degradation, other mechanisms, such as intracellular degradation mediated by other numerous cytosolic proteases and/or reduced mRNA translation, cannot be ruled out.

## Methods

### Cell cultures

Chinese hamster ovary (CHO-K1, CCL-61) and human hepatoma (Huh7, RCB 1366) cells were obtained from ATCC (American Type Culture Collection, Rockville, MD). Cells were cultivated in Dulbecco's Modified Eagles Medium (DMEM) (Cambrex BioScience, Verviers, Belgium) supplemented with 100 IU/ml penicillin, 100 μg/ml streptomycin, and 10% foetal calf serum (FCS) (Biowhittaker (TM), Luna, Belgium). Cells were incubated at 37°C in a humidified atmosphere with 95% air and 5% CO_2_.

### Site-directed mutagenesis and construction of the expression vectors

Total RNA from Huh7 cells was isolated using the ABI PRISM 6100 system (Applied Biosystems, Foster City, CA) following the manufacturer's procedure. Full-length PC cDNA was amplified by PCR with the following primers (forward primer, 5'-GAC GGC GAA CTT GCA GTA T-3' and reverse primer, 5'-ATC CCC CTC AAC ACA CAC AG - 3') (Eurogentec, Seraing, Belgium). The obtained cDNA was cloned into the pcDNA3.1/V5-His-TOPO vector (pPROC-WT) (Invitrogen, Carlsbad, CA). cDNA for the A267T PC variant was created using the Quick^®^Change Site-Directed Mutagenesis Kit (Stratagene, Amsterdam, Netherlands) according to the manufacturer's instructions. The primers were as follows (forward primer, 5'-CTG CAC CTG GCC CAG CCC ACC ACC CTC TCG CAG ACC ATA GTG CCC-3' and reverse 5'-GGG CAC TAT GGT CTG CGA GAG GGT GGT GGG CTG GGC CAG GTG CAG-3' (Eurogentec). The entire sequence of both PC cDNAs was confirmed using gene specific (Eurogentec) and vector (Invitrogen) primers.

### Transient transfections

For transient transfections, CHO-K1 and Huh7 cells were grown to 70-80% confluence in six-wells plates one day prior to the transfection. The transfections were performed with FuGENE-6^® ^Transfection Reagent (Roche, Mannheim, Germany) at a 1:6 ratio (DNA: FuGENE-6^® ^Reagent) following the manufacturer's instructions. 1 μg plasmid DNA was used per well. For each transfection reaction, empty vector was used as a negative control. The transfection efficiency was evaluated by co-transfection with pRcCMV/CAT plasmid (Invitrogen). The activity of chloramphenicol acetyltransferase (CAT) was measured using CAT-ELISA Kit (Roche) and no statistical significant differences (Mann-Whitney test) of CAT activities were observed in lysates from cells co-transfected with either pPROC-wt or pPROC-A267T (CHO-K1, *p *= 0.507 and Huh7, *p *= 0.186). The median and range of CAT activities (arbitrary units) in lysates were 15.1 (10.2-21.9) for pPROC-WT and 13.7 (11.1-17.5) for pPROC-A267T in CHO-K1 cells and 6.5 (3.9-8.6) for pPROC-WT and 5.9 (3.6-8.2) for pPROC-A267T in Huh7 cells_._

### PC expression measurements by ELISA

24 and 48 h after transfection, the culture medium was collected and the cells were washed three times with pre-chilled phosphate-buffered saline (PBS) before extraction in 800 μl of ice-cold RIPA lysis buffer containing a protease inhibitor cocktail (Sigma-Aldrich, St. Louis, MO). PC antigen concentrations in cell lysates and culture medium were measured using the Elisara Protein C Kit (Aniara, Mason, OH). The total protein concentration in cell lysates was measured by the Lowry Bio-Rad *D_C _*Protein Assay (Bio-Rad, Hercules, CA). PC antigen levels in cell lysates or culture medium were normalized against the total protein concentrations of the corresponding lysate samples. The results for Huh7 cells were normalized by subtracting the endogenous PC levels.

### Quantitative RT-PCR (qRT-PCR)

24 h after transfection the cells were washed with pre-chilled PBS and lysed in 800 μl Nucleic Acid Purification Lysis Solution (Applied Biosystems). Total RNA was isolated using the ABI PRISM 6100 System (Applied Biosystems) according to the manufacturer's procedure and quantified with a NanoDrop ^®^ND-1000 Spectrophotometer (NanoDrop Technologies, Wilmington, DE). 600-1200 ng of total RNA was reversely transcribed using the High Capacity cDNA Reverse Transcription Kit (Applied Biosystems) with random primers according to the manufacturer's procedure. PC mRNA levels were determined by the 7900 HT Fast Real-Time PCR System (Applied Biosystems) using a TaqMan^®^Gene Expression Assay for PC (Applied Biosystems) and TaqMan^®^Universal Master mix (Applied Biosystems) according to the manufacturer's instructions. As endogenous controls for normalization of the amount RNA in the reaction and for RT efficiency, assays for the glyceraldehyde-3-phosphate dehydrogenase (GAPDH) and the transferrin receptor (TFRC) cDNAs were used for Huh7 cells, and the TATA binding protein (TBP) and small subunit rRNA (18S) cDNAs for the CHO-K1 cells. The PC mRNA levels in Huh7 cells were normalized for the endogenous PC mRNA levels. All assays were from Applied Biosystems. Negative controls without cDNA were always included.

### N-linked glycosylation and Western analysis

Cell lysates for the deglycosylation reactions were prepared from cells transiently transfected to express WT-PC or A267T PC. The cells were harvested 48 h after transfection and washed with pre-chilled PBS followed by the addition of 800 μl of ice-cold RIPA lysis buffer containing a protease inhibitor cocktail (Sigma-Aldrich). The cell lysates and the corresponding media were then incubated with an endo-β-N-acetylglycosaminidase (Endo-H) enzyme (Roche) for 18 h at 37°C according to the manufacturer's instructions. Following deglycosylation, the samples were resolved on a 10% SDS-PAGE gel under reduced conditions and electroblotted onto a PVDF membrane (Millipore, Bedford, MA). After blocking in 5% (w/v) non-fat milk in TBS-T buffer (Bio-Rad) the membranes were incubated with rabbit anti-human antibodies against PC (1 μg/ml, Aniara) overnight at 4°C followed by an incubation with a horseradish peroxidase-conjugated goat anti-rabbit IgG antibody (0.1 μg/ml, Santa Cruz) for 1 h at room temperature. Proteins were visualized with the ECL Western blot detection kit (Pierce, Rockford, IL) on High Performance chemiluminiscence film (GE Healthcare, Buckinghamshire, UK).

### Degradation studies

To determine the intracellular half-lives of WT and A267T PC, CHO-K1 cells were transiently transfected with pPROC-WT and pPROC-A267T and grown for 24 h. The cells were then treated with CHX (50 μg/ml) (Sigma-Aldrich) in serum-free medium for various time-points. Intracellular PC antigen and total protein levels were determined as described above. To examine the potential degradation through the proteasomal and lysosomal pathways, transiently transfected cells were treated ± 10 μM lactacystin (Sigma-Aldrich) or 100 nM bafilomycin (Sigma-Aldrich) for 24 and 8 h, respectively. The agents were added 24 h post transfection. PC antigen and total protein levels in cell lysates were determined as described above.

### Confocal microscopy

CHO-K1 cells (1.1 × 10^5 ^cells/well) were grown on coverslips in a six-well plate and transfected with 1 μg of plasmid using the FuGENE-6 Transfection Reagent (Roche). Forty-eight h after transfection, the cells were fixed with 10% formalin solution (Sigma-Aldrich), followed by permeabilization in 0.1% Triton X-100 (Sigma-Aldrich) in PBS and blocking in 5% foetal bovine serum (FBS) (Biowhittaker (TM), Luna, Belgium). Immunostaining was performed with appropriate primary antibodies: rabbit polyclonal anti-PC (Aniara), mouse monoclonal anti-PDI (Stressgen, Ann Arbor, MI) or mouse monoclonal anti-GM130 (BD Biosciences, San Jose, CA) diluted in PBS with 5% FBS. Cy-2 and Cy-3-conjugated secondary antibodies were from Jackson Immunoresearch (West Grove, PA). The cells were mounted in Mowiol 4-88 (Merck Chemicals Ltd, Nottingham, UK) and analyzed using the laser scanning confocal microscope LSM 510 Meta (Carl Zeiss, Jena, Germany). Images were prepared with the ImageJ software (National Institute of Health, USA).

### Statistical analysis

All results were tested for the statistical significance with the non-parametric two-tailed Mann-Whitney and Wilcoxon tests. *p*-values <0.05 were considered statistically significant. GraphPad Prism version 5 (GraphPad Software, San Diego CA) was used for statistical analysis.

## Authors' contributions

LT carried out the cellular and molecular biology experiments, participated in the study design, interpretation of data and drafted the manuscript. JB performed the confocal microscopy analysis and edited the manuscript. NI and PMS participated in the design of the study and the interpretation of the data and edited the manuscript. KS participated in the interpretation of the data and edited the manuscript. GS participated in the design of the study, the interpretation of the data and helped to draft the manuscript. All authors have read and approved the final manuscript.
